# Renal Progenitor Cells Have Higher Genetic Stability and Lower Oxidative Stress than Mesenchymal Stem Cells during In Vitro Expansion

**DOI:** 10.1155/2020/6470574

**Published:** 2020-07-10

**Authors:** Elís Rosélia Dutra de Freitas Siqueira Silva, Napoleão Martins Argôlo Neto, Dayseanny de Oliveira Bezerra, Sandra Maria Mendes de Moura Dantas, Lucilene dos Santos Silva, Avelar Alves da Silva, Charlys Rhands Coelho de Moura, Antônio Luíz Gomes Júnior, Débora Cavalcante Braz, José Ricardo Freitas Costa, Yulla Klinger de Carvalho Leite, Maria Acelina Martins de Carvalho

**Affiliations:** ^1^Integrated Nucleus of Morphology and Stem Cell Research (NUPCelt), Center for Agrarian Sciences, Federal University of Piauí, Brazil; ^2^Federal Institute of Education, Science and Technology of Piauí, Brazil; ^3^Program in Biotechnology (RENORBIO), Federal University of Piauí, Brazil; ^4^Center for Health Sciences, Federal University of Piauí, Brazil; ^5^Center for Higher Studies of Caxias, State University of Maranhão, Brazil

## Abstract

In vitro senescence of multipotent cells has been commonly associated with DNA damage induced by oxidative stress. These changes may vary according to the sources of production and the studied lineages, which raises questions about the effect of growing time on genetic stability. This study is aimed at evaluating the evolution of genetic stability, viability, and oxidative stress of bone marrow mesenchymal stem cells (MSCBMsu) and renal progenitor cells of the renal cortex (RPCsu) of swine (Sus scrofa domesticus) in culture passages. P2, P5, and P9 were used for MSCBMsu and P1, P2, and P3 for RPCsu obtained by thawing. The experimental groups were submitted to MTT, apoptosis and necrosis assays, comet test, and reactive substance measurements of thiobarbituric acid (TBARS), nitrite, reduced glutathione (GSH), and catalase. The MTT test curve showed a mean viability of 1.14 ± 0.62 and 1.12 ± 0.54, respectively, for MSCBMsu and RPCsu. The percentages of MSCBMsu and RPCsu were presented, respectively, for apoptosis, an irregular and descending behavior, and necrosis, ascending and irregular. The DNA damage index showed higher intensity among the MSCBMsu in the P5 and P9 passages (*p* < 0.05). In the TBARS evaluation, there was variation among the lines of RPCsu and MSCBMsu, presenting the last most significant variations (*p* < 0.05). In the nitrite values, we identified only among the lines, in the passages P1 and P2, with the highest averages displayed by the MSCBMsu lineage (*p* < 0.05). The measurement of antioxidant system activity showed high standards, identifying differences only for GSH values, in the RPCsu lineage, in P3 (*p* < 0.05). This study suggests that the maintenance of cell culture in the long term induces lower regulation of oxidative stress, and RPCsu presents higher genetic stability and lower oxidative stress than MSCBMsu during in vitro expansion.

## 1. Introduction

The in vitro senescence of multipotent cells, such as mesenchymal stem cells (MSC) and renal progenitor cells (RPC), has been commonly associated with macromolecular damage, especially to DNA induced by oxidative stress [1]. These changes are most frequently identified in cultures with prolonged incubation time, from the fourth passage, which limits the possibility of clinical application given the intrinsic need for cell expansion in vitro to obtain the minimum therapeutic concentration of 10^6^ cells/mL [2, 3].

Contemporary studies have identified that early passages, until the third passage, maintain the intrinsic characteristics of prolificity, viability, plasticity, antioxidant, and immunomodulation of some cell lines [4–6]. However, these observations may vary according to the sources of production and the lines studied, which raises relevant questions about the effect of growing time on genetic stability [7–10].

Recently, some studies considered cell therapies using MSC and RPC for the treatment of kidney lesions [11–15]. The therapeutic application of these cell lines raises relevant debates regarding the potential risks inherent to the technique, as well as its efficacy resulting from idiosyncrasies such as plasticity, immunomodulation, mutagenesis, and DNA damage, among others, developed under long-term culture [16]. An alternative is the use of lines in the early passages until the third passage. However, the maintenance of genetic stability in MSC and RPC has not been comparatively elucidated, both between lines and in vitro incubation time. Moreover, it has been an option that the use of RPC could benefit the differentiation of this lineage into specific kidney cells and, consequently, therapy for kidney diseases [14, 15]. However, this hypothesis still lacks validation.

The genetic stability of stem cells can be evaluated using assays to measure oxidative stress, tests to determine the integrity of the DNA strand and by necrosis and apoptosis indexes, and growth curves during its expansion in culture [17–19].

Among the trials to assess DNA integrity, comet testing is often used to detect strand breaks, commonly caused by reactive oxygen species [17, 18, 20]. Besides, measurements of reactive substances of thiobarbituric acid (TBARS) as markers of lipid peroxidation, nitric oxide production by nitrite levels, and components of the enzymatic defense system, such as reduced glutathione (GSH) and catalase, are also often used as a simple, fast, and economical alternative for measuring oxidative stress in culture [4, 21, 22].

However, few studies comparatively evaluated genetic stability and oxidative stress between MSC and RPC during in vitro expansion. Given the persistence of these relevant doubts, this study is aimed at assessing the evolution of genetic stability, viability, and oxidative stress of MSC derived from bone marrow and RPC obtained from the renal cortex of swine (Sus scrofa domesticus) in passages of culture.

## 2. Materials and Methods

### 2.1. Experimental Design

This study was authorized by the ethics committee on the use of animals of the Federal University of Piauí, no. 269/16, according to the standards of the National Council for the Control of Animal Experimentation (COBEA/Brazil).

A completely randomized design is composed of two groups with three replications each, consisting of mesenchymal stem cells derived from bone marrow (MSCBMsu) and progenitor cells derived from renal cortex (RPCsu) of swine (Sus scrofa domesticus), and evaluated in duplicate at the P2, P5, and P9 passages for MSCBMsu and P1, P2, and P3 for RPCsu.

The choice of evaluation passages was based on the scientific literature [2, 3], and the experimental groups were submitted to viability tests by 3-(4,5-dimethylthiazol-2-yl)-2,5-diphenyltetrazolium bromide (MTT), apoptosis and necrosis assays, comet test, and measurements of TBARS, nitrite, GSH, and catalase.

### 2.2. Kinetics and Cell Viability Assay

The Integrated Center of Morphology and Stem Cell Research (NUPCelt) donated the MSCBMsu and RPCsu. The cells were thawed conventionally, according to Bezerra [23].

Cellular kinetics was evaluated by saturation growth curve assay, in duplicate, using MSCBMsu in P5 and RPCsu in P2, with 7.6 × 10^3^ cells/mL. The cells were thawed, and the initial viability was immediately evaluated by 3-(4,5-dimethylthiazol-2-yl)-2,5-diphenyltetrazolium bromide (MTT). The cells were seeded in 24 wells, each well randomly trypsinized and submitted to the new MTT assay in DMEM High Glucose® (Invitrogen™, USA) in the ratio of 1 : 9 every 72 hours, as described by Zhou et al. [24].

Briefly, after incubation with MTT in DMEM for five hours, the supernatant was aspirated, and the culture was washed with 200 *μ*L of dimethyl sulfoxide (DMSO) for 30 minutes and evaluated in a spectrophotometer at 550 nm.

### 2.3. Analysis of Apoptosis and Necrosis

The cells were trypsinized and washed to 10^6^ cells/mL with PBS and resuspended in buffer solution (1x).

Samples were centrifuged at 480g; then, 100 *μ*L of the solution was transferred to a tube and 5 *μ*L of PE Annexin V and 5 *μ*L 7-AAD of I were added using Annexin V-PE Apoptosis Detection Kit (BD Pharmingen™, no. 559763) with light agitation, then incubated for 15 minutes at room temperature (25°C) in the dark. With the complementary addition of 400 *μ*L of binding buffer in each tube, they were analyzed by flow cytometry within 1 hour.

### 2.4. Comet Test (Genetic Instability)

The trial was performed under alkaline conditions (pH > 13), according to Da Silva et al. [25]. Briefly, an aliquot (10 *μ*L) of 10% homogenate (pellet and sodium phosphate buffer, pH 7.4) was mixed with 95 *μ*L of 0.75% agarose, homogenized, and arranged on slides coated with 5% agarose. These were kept at 30°C for 20 minutes, immersed in lysis solution (2.5 M/L NaCl, 100 mM/L EDTA, 10 mM/L Tris-HCl, pH 10-12, 1% Triton X-100, and 10% DMSO), and packed in a dark environment at 4°C for 72 hours.

Subsequently, we performed electrophoresis using a buffer solution (30 mM/L NaOH and 1 mM/L of EDTA, pH 13) at 25 V and 300 mA at 30°C for 20 minutes, neutralized (400 mM Tris, pH 7.5) in three five-minute cycles. The slides were washed; dried; fixed in 15% (*w*/*v*) trichloroacetic acid, 5% (*w*/*v*) zinc sulfate, and 5% (*w*/*v*) glycerol solution; hydrated; and stained with the association of 0.2% (*w*/*v*) ammonium nitrate, 0.2% (*w*/*v*) silver nitrate, 5% (*w*/*v*) tungstosilic acid, 0.15% (*w*/*v*) formaldehyde, 5% (*w*/*v*) sodium carbonate, and 5% (*w*/*v*) sodium carbonate solutions, for 30 minutes at 37°C.

The evaluation followed the methods of Carvalho et al. [26] to calculate the damage frequency (DF) and the damage index (DI). DF is based on the number of cells with or without tail, and DI refers to the cells visually allocated in five classes according to the tail size (0 = no tails and 4 = tails of maximum length) in a single DNA damage score for each sample and consequently for each group studied, ranging from 0 (completely undamaged = 100 cells × 0) to 400 (maximum damage = 100 cells × 4).

### 2.5. Evaluation of Thiobarbituric Acid Reactive Substance (TBARS) and Nitrite Concentration

We performed the assay to measure TBARS reagents using a method described by Draper and Hadley [27]. In general, 10% homogenate was stirred with 10% trichloroacetic acid and 0.67% thiobarbituric acid solution, kept in boiling water for 15 minutes, and then cooled. The mixture was centrifuged with *n*-butanol at 480g for 5 minutes. The butanolic phase was measured in a spectrophotometer at 535 nm. Protein concentration was determined, according to Lowry et al. [28]. We expressed the results in nM/g of the pellet.

Nitrite concentration was measured according to the method described by Green et al. [29]. We added in a tube 500 *μ*L of Griess reagent and 500 *μ*L of 10% homogenate, reading on a spectrophotometer at 560 nm.

### 2.6. Determination of Reduced Glutathione Level (GSH)

We adopted Ellman's reaction assay (5,5′-dithiobis-(2-nitrobenzoic acid)), according to Sedlak and Lindsay [30].

In a test tube, we added 400 *μ*L of 10% homogenate, 320 *μ*L of distilled water, and 80 *μ*L of 50% trichloroacetic acid. The material was stirred and centrifuged at 960g for 15 minutes, and the supernatant was diluted in 800 *μ*L of 0.4 M Tris-HCl buffer, pH 8.9, and 20 *μ*L of 0.01 M DTNB (5,5′-dithiobis-(2-nitrobenzoic acid)), and the absorbance was evaluated after 1 minute at 412 nm.

### 2.7. Determination of Catalase Activity

We used the method described by Chance and Maehly [31]. The reaction medium was prepared with H_2_O_2_ (18 mL), 1 M Tris-HCl buffer, 5 nM EDTA, pH 8.0 (1.0 mL), and H_2_O (0.8 mL). An aliquot (980 mL) was mixed with 20 *μ*L of 10% homogenate and incubated at 37°C; then, we read the absorbance levels every 1 minute up to 6 minutes at 230 nm. The absorbance of the reaction medium, without homogenate, was considered white. Protein concentration was determined, according to Lowry et al. [28].

### 2.8. Statistical Analyses

The data were submitted to analysis of variance (ANOVA), followed by the post hoc Newman-Keuls test, using GraphPad Prism 7.0 software (San Diego, CA, EUA). Cell cultures were compared to each other between the P2, P5, and P9 passages for MSCBMsu and P1, P2, and P3 for RPCsu. Significance was adopted to reject the 5% null hypothesis.

## 3. Results

The curve elaborated by the MTT assay showed an average viability of 1.14 ± 0.62 and 1.12 ± 0.54, respectively, for MSC and RPC.

**Figure 1:**
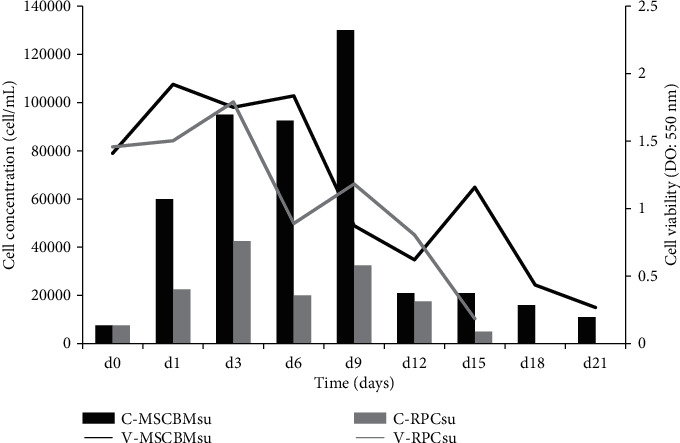
Relationship between mean concentration and viability observed in the cell kinetics assay, performed by the MTT colorimetric test of mesenchymal stem cells (MSCBMsu) and swine renal progenitor cells (RPCsu). C-MSCBMsu: MSCBMsu concentration; C-RPCsu: RPCsu concentration; V-MSCBMsu: MSCBMsu viability; V-RPCsu: RPCsu viability.

The MSCBMsu demonstrated logarithmic kinetics from the initial day to the ninth day, showing an evident decay phase from the 12th day. Diametrically, the RPCsu showed a logarithmic phase, only until the third day. Then, it presented a decay phase interspersed with a short logarithmic phase on the ninth day, when the decay was reestablished (Figure 1).

**Figure 2:**
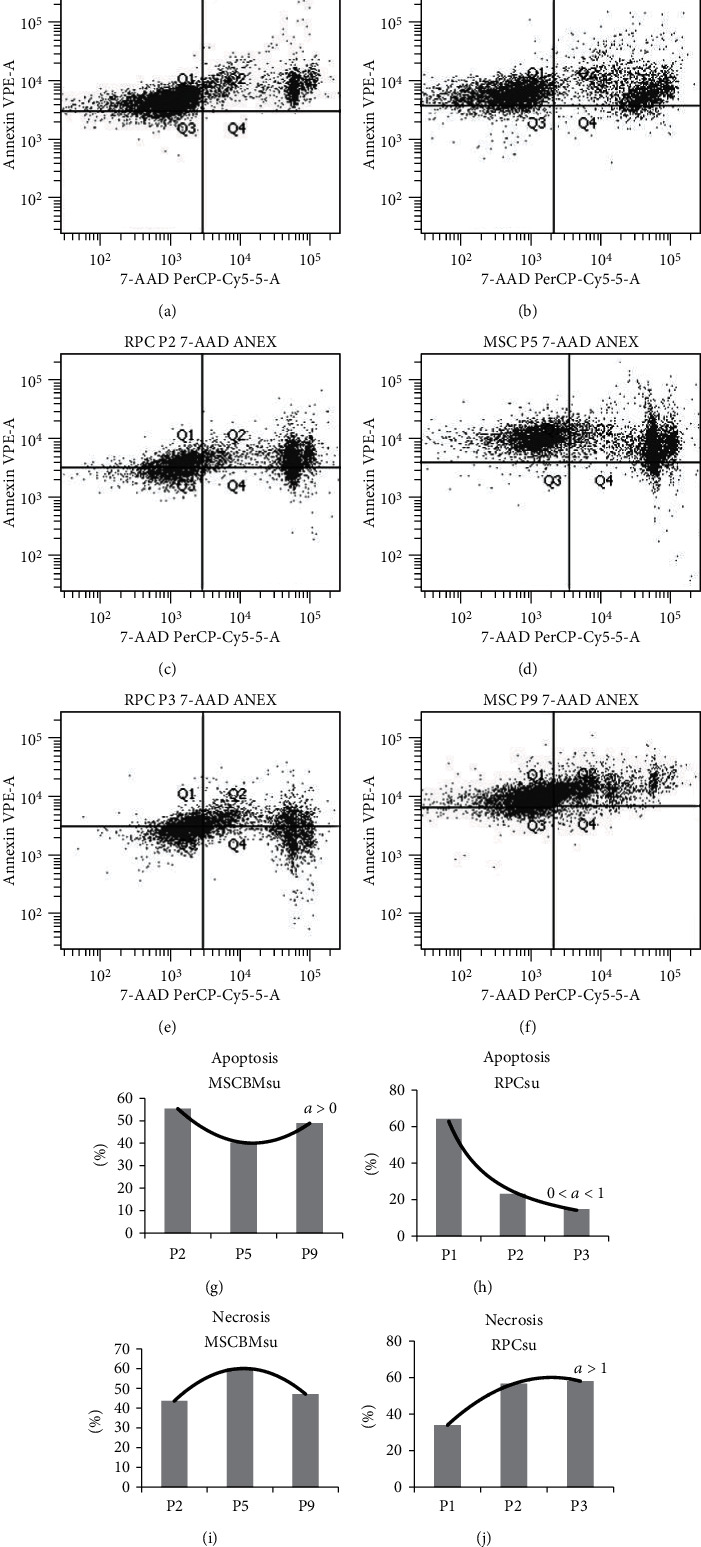
Analysis of the expression of Annexin V PE/7-AAD by flow cytometry for apoptosis/necrosis marking in different passages (P) of renal progenitor cells (RPCsu) ((a) P1, (c) P2, and (e) P3) and mesenchymal stem cells from bone marrow (MSCBMsu) swine ((b) P2, (d) P5, and (f) P9) with a graphical representation of the percentage of cells in apoptosis ((g) MSCBMsu, (h) RPCsu) and necrosis ((i) MSCBMsu, (j) RPCsu).

The percentages of apoptosis between the MSCBMsu and RPCsu lines presented polynomial distribution, identifying positive parabolic (*a* > 0) and negative exponential (0 < *a* < 1) curves, respectively (Figures 2(g) and 2(h)). Regarding the percentages of necrosis, it evidenced a curve in Gaussian-type behavior for MSCBMsu and of the positive exponential type (*a* > 1) for RPCsu (Figures 2(i) and 2(j)).

Comparing the percentages of apoptosis and necrosis within the same lineage, we observed that, for MSCBMsu, the rate of apoptosis was irregular, reaching maximum levels in P2 and P9 and minimum levels in P5. Diametrically, the percentages of necrosis for this lineage showed ascending behavior. For the RPCsu lineage, the rates of apoptosis showed descending expression, while the rates of necrosis were irregular, reaching minimum levels in P1 and P3 and maximum levels in P2 (Figure 2).

The comet assay identified no significant differences (*p* > 0.05) between the frequency of DNA damage but with significance (*p* < 0.05) for the DNA damage index among the studied lineages.

**Figure 3:**
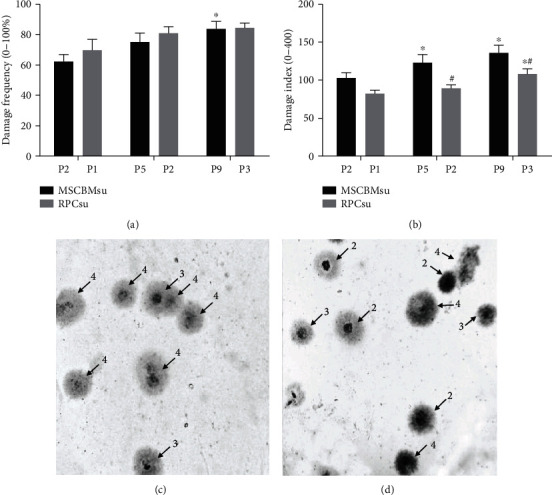
Assessment DNA damage of bone marrow mesenchymal stem cells (MSCBMsu) and renal progenitor cells (RPCsu) in the different culture passages. Frequency DNA damage (a), index DNA damage (b), severity classification in MSCBMsu (c), and severity classification in RPCsu (d). ∗ and #: significant differences between the passages, compared to the initial passages, of each lineage. ANOVA and Newman-Keuls: *p* < 0.05.

We observed that the P1 and P2 passages presented low frequency and DNA damage index for RPCsu and MSCBMsu, respectively. Over the course of the passages, the rate of DNA damage of the MSCBMsu increased significantly in P9 (*p* < 0.05). The DNA damage index showed higher intensity among the MSCBMsu (*p* < 0.05) in the P5 and P9 passages (Figures 3(a) and 3(b)).

DNA damage assessment identified 75% of severe damage (grade 4) and 25% of moderate damage (grade 3) in MSCBMsu cultures. For RPCsu cultures, the results identified 44.4% of mild damage (grade 2), 22.3% of moderate damage (grade 3), and 33.3% of severe damage (grade 4) (Figures 3(c) and 3(d)).

**Figure 4:**
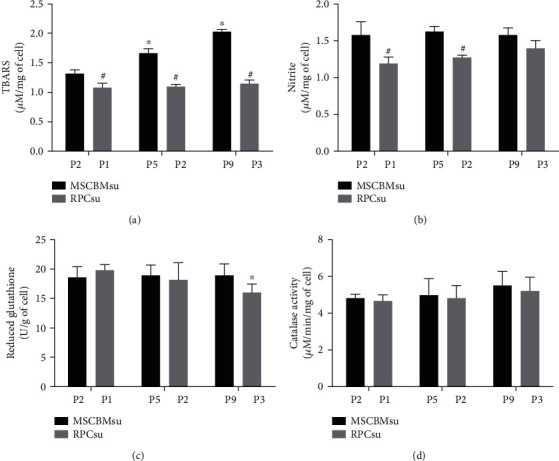
Measurement of TBARS levels (a), nitrite (b), reduced glutathione (c), and catalase (d) in bone marrow mesenchymal stem cells (MSCBMsu) and renal progenitor cells (RPCsu) in the different culture passages. ^∗^Difference between the passages (compared to initial passages) of each lineage; ^#^Difference between the groups of each passage. ANOVA and Newman-Keuls: *p* < 0.05.

The evaluation of oxidative stress did not identify significant variations (*p* > 0.05) of TBARS levels between the different passages of the RPCsu lineage. The mean values were 1.08 ± 0.08 nM, 1.10 ± 0.04 nM, and 1.15 ± 0.07 nM in the P1, P2, and P3 passages, respectively. However, there was significant variation (*p* < 0.05) of this marker between the RPCsu and MSCBMsu lines, presenting the last highest variations, 1.32 ± 0.06 nM, 1.67 ± 0.07 nM, and 2.03 ± 0.04 nM, in the same passages, respectively (Figure 4(a)).

The evaluation of nitrite values identified significant variation (*p* < 0.05) only between the lines, in the P1 and P2 passages, with the highest averages displayed by the MSCBMsu lineage (Figure 4(b)).

The measurement of antioxidant system activity, by GSH and catalase, identified significant differences (*p* < 0.05) only for GSH values, in the RPCsu lineage, in P3. The means presented by the antioxidant system were high, denoting positive activity (Figures 4(c) and 4(d)).

## 4. Discussion

This study is the first to evaluate the correlation between DNA damage and oxidative stress in mesenchymal stem cells and renal progenitor cells in culture. Previous studies using these lineages evaluated general characteristics of cell cultivation and tissue repair capacity [23, 32]. However, as the in vitro cellular behavior and, consequently, its therapeutic potential in vivo are subordinated to cellular resilience and metabolic adaptation capacity to possible molecular injuries, this study is aimed at seeking these answers in a frontier area of knowledge.

Initially, we identified that the MSCBMsu presented kinetics in culture higher than the RPCsu. The growth curve model adopted by saturation subjected cultures to relevant metabolic stress, since it did not use rebound to limit cell expansion and, consequently, exhaustion of the culture medium. Nevertheless, MSCBMsu showed a logarithmic increase over nine days before showing a reduction in cell concentration (Figure 1). This characteristic is not uncommon among mesenchymal stem cell lines, and studies with bone marrow, adipose tissue, and dental pulp lineages have commonly demonstrated similar evidence [33–35]. This observation raises the possibility that this group of somatic stem cells may present relevant metabolic adaptability.

The maintenance of the logarithmic growth of MSCBMsu in this study did not negatively influence the viability index. The MSCBMsu showed an average variation also higher than the RPCsu throughout the logarithmic phase (Figure 1). These data demonstrate a thriving and probably stable culture until the ninth day of cultivation. From then on, the decay of kinetics and viability reflected the exhaustion of nutrients from the culture medium, resulting from the prolonged total confluence.

Although the RPCsu exhibited lower growth kinetics, the viability index remained similar to those of the MSCBMsu, denoting a metabolically active culture (Figure 1). Corroborating this proposition, studies with this lineage [23, 36] describe shorter cellular kinetics due to a possible in vitro immaturity, which restricts long-term expansion [37]. It is believed, therefore, that perhaps this is an intrinsic characteristic of RPCsu in culture. The possibility of the therapeutic use of this line would require a greater number of renal tissue samples for isolation and expansion in order to obtain adequate concentrations for use in preclinical and clinical trials. Contemporary studies used minimum cell concentration ranging from 2 to 5 × 10^6^ RPC/mL in clinical trials [38–40].

The apoptosis and necrosis tests indirectly corroborated the observations of the kinetics and viability tests, with the proliferation behavior and cell death following the variations of the growth curves for each lineage.

In general, the RPC showed lower rates of apoptosis and necrosis than the MSCBMsu after long-term incubation.

The MSCBMsu exhibited higher rates of logarithmic expansion on days 3, 6, and 9, with the highest apoptosis rate in the second passage, corresponding to the sixth day (Figures 2(b), 2(d), 2(f), and 2(g)). The increase in apoptosis rate may be related to the rise in the mitotic rates of cell culture. This relationship has been described in contemporary studies that investigate the increase in apoptosis rates in expanding cell cultures [41, 42]. This is a relevant mechanism of homeostasis in mammalian cells, during which formed apoptotic bodies are phagocytized by dendritic cells, favoring the transfer of trophic factors between cells and, consequently, cellular communication [43]. This mechanism, therefore, supports cell renewal, allowing continuous cell expansion [44]. Such syllogism has been defended by the demonstration, in classical studies, that the absence of apoptosis in mammalian cell cultures predisposes to the development of autoimmune disorders [45, 46].

Similarly, the RPCsu exhibited similar behavior, with a higher rate of apoptosis in the first passage (Figures 2(a), 2(c), and 2(h)), corresponding to the early three days of culture, which occurs with the peak of logarithmic expansion on the third day. The lowest rates of apoptosis from the second pass, when compared to the MSCBMsu, are probably due to the end of the logarithmic expansion phase and subsequent decay of the crop (Figure 1).

Necrosis rates in the cultivation of the MSCBMsu lineage were lower than the apoptosis rates in the second passage, with subsequent elevation in a fifth passage corresponding to the 15th day of cultivation, in which culture already presented kinetic decay. Similarly, necrosis rates of the RPCsu lineage were also lower than the apoptosis rates, at first passage, with elevation after the sixth day of cultivation. Similar results have been described [23, 33, 34]. The increase in these rates is probably due to exhaustion of the culture medium due to the large cellular confluence, inducing necrosis [33].

The data obtained for the index and frequency of DNA damage indicated low percentages in the initial passages, corresponding to three and six days of culture, respectively, for both lineages. These variables showed significant elevation only between the following passages (Figure 3). These results followed the pattern of in vitro behavior of the lineages studied in this research, which showed a reduction in kinetics, viability, increased necrosis, and, consequently, increased index and frequency of DNA damage after the third (P1) and ninth (P3) day, for the RPCsu and MSCBMsu lineages, respectively.

The behavior of the variables, index, and frequency of DNA damage allows the inference of direct correlation between them and the establishment of the culture exhaustion phase. A contemporary study raised this hypothesis earlier, describing increased DNA damage as strongly correlated to in vitro culture expansion time [18]. These authors observed that the highest rates of DNA damage were concentrated between the fourth and sixth passages, considered “late” passages, corresponding to the 12th and 18th days of cultivation.

Besides, intrinsic factors have also been incriminated as predictors of this in vitro behavior [7, 8]. A contemporary study suggested that MSCBMsu when in early passages would be more resistant to irradiation or induced DNA damage, mediated by polyenzyme (ADP-ribose) polymerase-1 (PARP-1) and ATM gene [47]. This can probably be considered one of the arguments to the recommendation of the therapeutic use of MSCBMsu, preferably in initial passages [6].

Oxidative stress assays showed that the RPCsu lineage presented lower lipid peroxidation indexes and nitrite production when compared to MSCBMsu (Figure 4). However, the means of TBARS measurements for both lines remained below the values referenced in previous studies of 3.31 *μ*M ± 0.38 [48, 49]. This observation suggests that, although RPCsu has lower rates of lipid peroxidation than MSCBMsu, in general, in this study, this oxidative change occurred at a more moderate intensity than that observed in other mammalian cells.

Nevertheless, the levels of the GSH antioxidant system and catalase were elevated for both lineages, denoting positive activity (Figure 4). It seems evident to us that the preservation of the antioxidant system may have contributed to the lower rates of lipid peroxidation and nitrite production, as well as the lower average of DNA damage index and frequency, observed previously in this study. Previous studies had already described such in vitro behavior, in which stem cell lineages in early passages exhibited high resistance to necrosis induced by oxidative stress, mediated by the cellular capacity to reduce intracellular production of reactive oxygen species, as well as preserve the constitutive expression of the antioxidant system [4, 50].

In this study, in general, it was identified that the increase in cell culture time and, consequently, the number of passages induced the increase in the means of lipid peroxidation and DNA damage, promoting genetic instability and cell necrosis, for both lineages. However, comparatively, the RPCsu exhibited lower averages, suggesting that in this lineage idiosyncratic factors that favor the low rates of oxidative stress may participate. On the other hand, the shorter logarithmic expansion time of this lineage, when compared to MSCBMsu, theoretically, could also induce the low oxidative stress rates reported.

The relation between in vitro cell expansion time and oxidative stress has been previously described as resulting from the marked production of EROS damaging proteins and DNA and inducing replicative senescence, known as premature senescence caused by stress [51].

We believe that such data, as well as the hypotheses listed in this discussion, may contribute to future clinical trials, both in the choice of cell lineage and in the time of culture for therapeutic administration. It seems evident to us that there is a directly proportional correlation between incubation time and oxidative stress. This fact may mitigate the relevant doubts about the therapeutic efficacy of these lines, as well as contribute to support the possible review of the International Society of Cell Therapy of the maximum limit of allowed passages for cultures intended for clinical application.

## 5. Conclusion

This study is the first to evaluate the correlation between DNA damage and oxidative stress in the RPCsu and MSCBMsu lineages, in which an inversely proportional relation was identified between growing time and cell viability and directly proportional between growing time, necrosis, lipid peroxidation, and DNA damage.

The results suggest that the maintenance of cell culture in the long term, in general, induces lower regulation of oxidative stress, with subsequently cellular senescence. This fact may be considered predictive of the maintenance of genetic stability in cultures until the third passage and, consequently, more excellent clinical safety and therapeutic effectiveness.

The comparative analysis between the lineages indicated that the RPCsu presented higher genetic stability and lower oxidative stress than the MSCBMsu during in vitro expansion and may be considered preferred for the treatment of kidney diseases.

## Data Availability

The data used to support the findings of this study are available from the corresponding author upon request.
